# Illusion of influence? Public participation in the preparation of climate policies in Finland

**DOI:** 10.1007/s13280-025-02316-4

**Published:** 2025-12-03

**Authors:** Anni Turunen, Suvi Huttunen

**Affiliations:** 1 Societal Change, Finnish Environment Institute, Survontie 9A, 40500 Jyvaskyla, Finland; 2 Department of Social Sciences and Philosophy, University of Jyväskylä, Jyvaskyla, Finland; 3 Societal Change, Finnish Environment Institute, Latokartanonkaari 11, 00790 Helsinki, Finland

**Keywords:** 4Ds of participation, Climate policy, Engagement, Environmental governance, Participatory collective, Public participation

## Abstract

Calls for acceptable climate policies render public participation central to climate policy planning. However, implementing participation remains challenging and often limited. To foster more effective and meaningful participation, it is essential to understand how participatory practices and efforts to develop them function in real-world policy processes. We develop this understanding by applying a systemic and relational approach that focuses on public participation as a collective and contextual phenomenon and integrate this with a normative 4D approach for evaluating participation through its capacity to advance dialogue, diversity, deliberation, and to allocate decision-making power. We empirically examine public participation in the preparation of Finland’s national climate policies from the perspective of government officials. Our findings show the usefulness of examining participation systemically and including its evaluation. In the Finnish context, the role of decision-making power in the participatory collectives remains weak and requires further attention.

## Introduction

Addressing the climate crisis requires urgent action from governments responsible for developing and implementing related policies (Gollata et al. 2021). Climate issues permeate all aspects of life and concern questions of our existence, making public participation essential for meeting climate targets (Albrecht et al. 2022). Citizens have a fundamental right to be involved in the governance of these matters (Sprain 2016). Citizen involvement in climate policy planning, development and implementation can generate legitimacy and acceptability for climate policies (Raman and Pearce 2020; Perlaviciute 2022) and improve their quality and effectiveness (Arango et al. 2024). For citizens, participation can foster learning and commitment to behavioural change (Ryan et al. 2023). Furthermore, public participation is seen as a way to mitigate the influence of political and economic elites (Galván Labrador and Zografos 2024). The benefits of public participation are endorsed by democratic governments. For example, the European Green Deal (2019) recognises the importance of active citizen involvement in designing and implementing successful climate policies. Overall, effective public participation is a prerequisite for addressing the climate crisis.

However, public engagement is not simple and can even be counterproductive (Carrick et al. 2023). Findings on the ability of participation to deepen democracy and bring about significant policy changes are conflicting (Bherer 2010). Participation is often unilateral with minimal citizen influence (Galende-Sánchez and Sorman 2021; Kinnunen 2021). Citizens’ capacities and wishes regarding participation vary (Murunga et al. 2024) and people do not necessarily want to participate or seek more power (Perlaviciute 2022). Climate issues are often framed as technical and requiring specialised knowledge, making public participation even more difficult. Strong expert involvement and reliance on scientific knowledge can narrow policy options and limit public deliberations (Raman and Pearce 2020; van Beek et al. 2024).

There is a need to develop better approaches and methods for public participation to reach its promises in the climate context. Rather than improving individual participatory methods, a systemic approach is needed to consider diverse contexts and actors (Braun and Könninger 2018; Chilvers and Kearnes 2020; Murunga et al. 2024). Recent developments in applying constructivist and relational perspectives from the Science and Technology Studies (STS) to public participation present promising avenues for understanding the diversity and complementary nature of public participation attempts (Chilvers and Longhurst 2016; Braun and Könninger 2018). Despite its promise, a participatory collective approach has not been applied in the policy planning context. Given its descriptive nature, adopting a more evaluative approach with normative criteria can help to address the functioning of the engagement efforts in generating acceptability. Hence, we make a novel contribution by combining the participatory collective approach with the 4Ds of participation approach proposed by Perlaviciute (2022), to distil the core themes needed for well-functioning public engagement.

To enhance the novelty, we examine public participation from the perspective of government officials, which is rarely done but important, as government officials are key intermediaries in policymaking (Michels and De Graaf 2010). They influence how policies are shaped and communicated to the public (Shaw et al. 2018), as well as the scope and methods of public participation in policy planning and the adoption of its outcomes (Kiss et al. 2022) highlighting the need to address participation from the administrative viewpoint (Gourgues et al. 2021). Empirically, we focus on national-level climate policy preparation in Finland involving multiple policies. A national-level examination is necessary, as norms of public participation should account for national contexts (Fritz and Koch 2019). Many studies (e.g. Cattino and Reckien 2021; Wells et al. 2021) focus on participation in local climate policies, but research on public participation in national-level climate policies has emerged only recently (e.g. Akerboom and Craig 2022; Albrecht et al. 2022). Although there are studies looking into national deliberative fora such as citizens’ conventions (Galván Labrador and Zografos 2024), we are not aware of other studies systematically examining participation in the climate policy of a single country across more than one policy and participation method.

Finland is known for its ambitious climate policy, strengthened during Marin’s government (2019–2023). The government reformed all major national-level climate policies and attempted to improve public participation in policy planning by experimenting with various citizen and stakeholder engagement methods. Alongside fostering the legitimacy of climate policies, the intention was to enhance democracy (Ministry of Justice 2022) and address observed challenges in public participation in Finland, including low participation in decision-making and low belief in citizens’ abilities to influence decision-making (Jämsén et al. 2022). Although still relatively high, trust in government and the political system is decreasing (OECD 2021). In Finnish climate policy, public opinion has had weak congruence with actual decisions (Kinnunen 2021). The engagement attempts in climate policy preparation form a useful arena for examining the functioning of different participatory collectives and provide lessons for improving public participation at the national level in other contexts.

To examine public participation in Finnish climate policy, we focus on three key policy preparation processes from 2019 to 2022: the Finnish Climate Change Act (hereafter Climate Act), the Climate and Medium-term Climate Change Policy Plan (hereafter Policy Plan), and the Climate and Energy Strategy (hereafter Strategy). Using a combination of the participatory collectives and the 4Ds of participation approaches, we pose two main questions:

1. What kind of participatory collectives emerge from the public participation efforts in Finnish climate policy preparation?
2. What positive and negative effects can be identified in relation to public participation processes?

The study seeks to provide a deeper understanding of how public participation is implemented in national-level climate policies, and what lessons can be learned to improve participation in order to generate more acceptable climate policies.

## Combining systemic and normative perspectives

### Public participation in climate policy

**Fig. 1:**
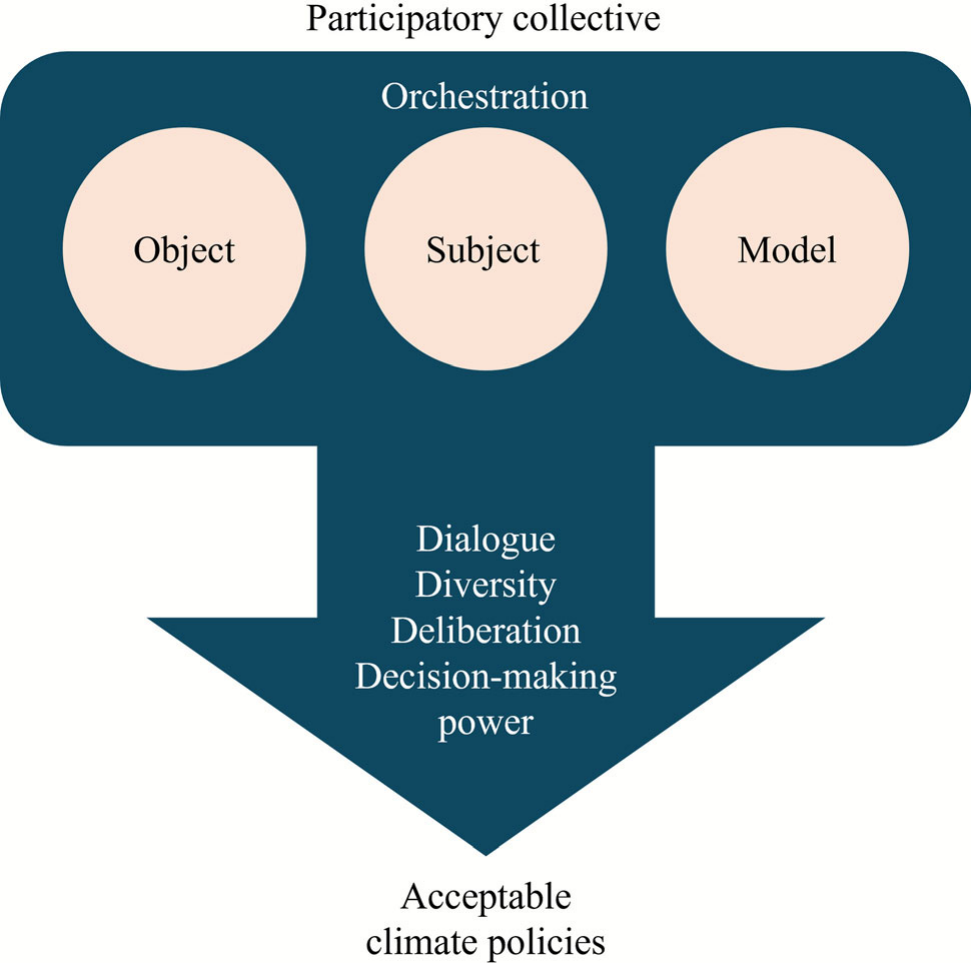
The theoretical background combines systemic and normative approaches to public participation to examine participatory collectives in the climate policy preparation and evaluate their impact on building dialogue, diversity, deliberation, and decision-making power for citizens

Public participation in climate policy includes participation in climate-related decision-making and participation in actions aimed at reducing greenhouse gas emissions in everyday life (Whitmarsh 2021). In this study, we focus on the former, and examine the administrative perspective on participation, the means and aims of getting people involved in policymaking. Research on public participation in the context of climate policy has focused on improving democracy, the practical benefits of public participation, and citizens’ possibilities to participate (Jodoin et al. 2015). Public participation is usually considered through distinct and one-off methods (Chilvers 2024) and focusing on identifying best practices (Chilvers and Kearnes 2020). However, recent years have seen the emergence of new directions drawing from STS (Braun and Könninger 2018; Chilvers and Kearnes 2020; Chilvers 2024). This involves understanding participation as relational—created through interactions and shaped by structures and actors (e.g. Fritz and Binder 2018). This systemic approach, focusing on participatory collectives, counters the idea that public participation is a detached and top-down event where the main problem lies with citizens rather than institutions (Braun and Könninger 2018). The approach can foster inclusivity by recognising public participation broadly, emphasising its collective aspects, and opening discussions on the transformative potential of public participation. However, the approach can be challenging to implement. It might also overemphasise the process at the expense of outcomes (Chilvers and Kearnes 2020), and be blind to institutional political action and power relations in co-production (Delvenne et al. 2024). This makes it fruitful to combine the systemic and relational approach of participatory collectives with the normative 4Ds of participation by Perlaviciute (2022), which pays attention to the outcomes and effects of public participation. Thus, it is possible to view participation both as a system and in terms of its impacts (Fig. 1).

### Participatory collective

According to the participatory collective approach, public participation is an emergent phenomenon, consisting of subject, object, model, and orchestration—mutually shaping and creating public participation (Chilvers and Longhurst 2016). Participation is not pre-defined or precise, but rather co-produced through diverse collective practices (Chilvers 2024). In these collectives, the *object* of participation is the issue around which participation is formed (Chilvers and Longhurst 2016), e.g. climate measures. The object can also be seen as the reason for engagement, e.g. building acceptability. The *subject* of participation refers to the participants, e.g. citizens (Chilvers and Longhurst 2016). In the context of policymaking, this means the target group for participation. The *model* of participation refers to the procedural forms of participation, which can be top-down—initiated by the administration, such as a citizens’ jury—or based on citizens' own actions and initiatives, both in influencing policies and in implementing climate-friendly actions in practice (Chilvers and Longhurst 2016). In the policymaking context, this refers to the different activities presented to the public to engage them in influencing the policy. *Orchestration* refers to the ways actors are brought into a specific participatory collective. This can be institution-driven, e.g. ministry-invited workshop, or more informal, e.g. citizen-initiated participation. Orchestration also describes the material processes, tools, and technologies through which a participatory collective is maintained. Beyond the factors bringing a participatory collective together and keeping it functioning, there are also exclusions, and both internal and external resistance within these collectives (Chilvers and Longhurst 2016).

The participatory collective approach helps to clarify how different factors together create public participation. By focusing on collectives and their constitution, the approach enables comparisons between various policy processes and participatory practices (Chilvers and Longhurst 2016). However, it offers no normative guidance for assessing the functioning of public participation or improving its outcomes.

### The 4Ds of participation in climate governance

The 4Ds approach presents a useful framework for evaluating public participation, as it is specifically created for analysing public participation in climate decision-making. The 4Ds provides clear normative standards for identifying the factors increasing the acceptability of climate policies, focuses on the outcomes of public participation, and allows for the assessment of both the positive and negative impacts of participation (Perlaviciute 2022). Furthermore, it recognises participation as a dynamic and emergent process, co-created among participants. Focusing attention on the normative framework can make responsible parties more aware of the impacts of participation and thereby prevent them from engaging the public in ways that result in, e.g. exclusion rather than inclusion. The 4Ds consist of dialogue, diversity, deliberation, and decision-making power, and their counterparts: exclusion, polarisation, and fake participation.

*Dialogue* refers to the facilitation of open and meaningful exchange of thoughts between participants (Perlaviciute 2022). Dialogue is essential for identifying mutual benefits and breaking down barriers hindering cooperation (Ansell and Gash 2008). In climate governance, dialogue has been fostered through methods that allow policymakers to hear first-hand information about how communities are impacted, and citizens to discuss local problems and solutions (Hurst et al. 2022). Dialogue involves two-way engagement and differs from the one-way transfer of information from citizens to policymakers via instruments such as surveys (Ainscough and Willis 2024).

*Diversity* refers to engaging participants with diverse backgrounds and perspectives, and aims to promote a more comprehensive and inclusive decision-making process. Democracy requires diverse involvement, which can enhance the quality and fairness of decision (Perlaviciute 2022), as well as support acceptable and effective climate mitigation (Cattino and Reckien 2021). Exclusion occurs when certain groups or voices are marginalised and not given the chance to participate (Perlaviciute 2022). In practice, in climate planning, the scope of participants is often too limited (e.g. Jetoo 2019).

*Deliberation* concerns informed discussions among participants, enabling them to consider various alternatives (Perlaviciute 2022). Deliberation is important for democracy and the acceptability of policy processes (Driver et al. 2018). It can be an effective way to govern polarised issues, such as climate change, and can foster agreement around proposed solutions (Ghimire et al. 2021). Deliberative democratic processes can provide a voice to citizens and create momentum around climate action, but their impact on policymaking may be indirect or unsupportive (Pettenger 2016). Polarisation occurs when participation leads to increased division. People do not always accept viewpoints that differ from their own, and may be unwilling to compromise, making it harder to reach agreement on policies (Perlaviciute 2022).

*Decision-making power* refers to the ability of participants to influence the decision-making process, ensuring that their input is genuinely considered (Perlaviciute 2022). While participatory processes can impact decision-making, various factors determine the extent of citizen power (Young and Tanner 2023). Fake participation occurs when people are included in decision-making superficially, without any real power to influence the decisions made (Perlaviciute 2022). Climate policies often fail to fully integrate the outcomes of public participation in their formulation and implementation, thereby perpetuating existing power relations and institutionalised truths (Mendonça et al. 2023).

## Materials and methods

Officials from the Ministry of the Environment and The Ministry of Economic Affairs and Employment are responsible for national climate policy preparation. Interviewing the responsible officials allowed us to understand how, why, and what kind of public participation took place. Public and stakeholder engagement was documented in publicly available documents. While focusing on public participation, we also acknowledged stakeholder and expert participation—even though not public participation—as they were involved in the processes, and studying a participatory collective without acknowledging them would be incomplete. Here, stakeholders refers to the representatives of organised groups, and citizens or the public to individual members of society (Kahane et al. 2013).

Our data consists of eight thematic interviews with government officials and 29 policy documents (Table 1). We selected all officials centrally involved in the preparation process and obtained their contact information from public sources. The number of interviewees represents a comprehensive sample of the officials involved in the preparations, as the work was carried out by a small group and the data reached saturation. The interviews were conducted online via Microsoft Teams in 2023, lasting an average of 55 min. According to the Finnish National Board on Research Integrity TENK, ethical assessment was not required for the study. We sent a research notification and privacy notice to interviewees in advance. In the privacy notice, it was acknowledged that “Due to the nature of the topic, it is possible that dedicated persons will recognise the interviewees”. The interviewees signed a consent form agreeing to this. Passages from the interviews are marked in the text as O1, O2, etc., referencing individual officials.

**Table 1:** The participation processes and the analysed data, including interviews and policy documents

**Climate act**	
**Output**	
Climate Act 423/2022	
**Interviewees**	
Official 1, Official 2, Official 3, Official 4	
**Engagement**	
*Activity*	*Related document(s) if available and the reference to it in references*
Citizen survey	Results of the Citizen Survey (Tampere University, n.d.)
Library tour	Summary of Citizen Hearings (Ministry of the Environment, n.d.-a)
Stakeholder hearings for agriculture and land use sectors, business, municipalities and legal scholars	Summary of Stakeholder Hearings (Ministry of the Environment, 2021d)
Hearings for Youth Agenda and UN Youth	Summary of Citizen Hearings (Ministry of the Environment, n.d.-a)
Time Out discussions	Summary of Citizen Hearings (Ministry of the Environment, n.d.-a)
Sámi Parliament negotiations 1–3	Minutes of the Sámi Parliament Negotiations (Ministry of the Environment, 2020, 2021a, 2021e)
Online citizen hearing	Report on the results of the online citizen hearing on the climate law (Aitamurto and Vento 2021)
Climate policy round table	
The State Youth Council meeting	
Hearing for Sámi youth	Summary of Citizen Hearings (Ministry of the Environment, n.d.-a)
Youth Academy workshops	
Skolt Saami Village Committee meeting	Minutes of the Skolt Saami Village Committee Hearing (Ministry of the Environment 2021c)
Statement hearings	Summary of Statement Hearings (Ministry of the Environment, 2021f)
Hearing for Extinction Rebellion	
**Climate Change Policy Plan**	
**Output**	
Medium-term Climate Change Policy Plan: Towards a carbon–neutral society in 2035 (Ministry of the Environment 2022)	
**Interviewees**	
Official 1, Official 5, Official 6	
**Engagement**	
*Activity*	*Related document(s) if available*
Youth survey	Column on engagement of the youth (Heiskanen 2021)
Equality workshop 1–2	Summary of Equality Workshops Results (Ministry of the Environment, n.d.-e)
Student and researcher hearing in English	
Hearing for clients of child welfare aftercare services	
Kick-off webinar	
Citizen survey	Summary of Multiple-Choice Questions from Citizen Survey (Ministry of the Environment, n.d.-c) Report on Open Responses from Citizen Survey (Repo and Matschoss 2021)
Sámi Parliament negotiations	Minutes of the Sámi Parliament Negotiation (Ministry of the Environment 2021b)
Stakeholder workshop	Summary of Stakeholder Workshop Session (Ministry of the Environment, n.d.-d)
Citizen council	Citizen Council Declaration (Keskipitkän aikavälin ilmastosuunnitelman kansalaisraati, n.d.) Final Report of the Citizens’ council on Climate Actions (Kulha et al. 2021)
Climate policy round table	
Skolt Saami Village Committee meeting	Minutes of the Skolt Saami Village Committee Hearing (Ministry of the Environment 2021c)
Youth Academy workshop for students	
Statement hearings	Summary of Statement Hearings (Ministry of the Environment, n.d.-b)
**Climate and Energy Strategy**	
**Output**	
Carbon neutral Finland 2035—national climate and energy strategy (Ministry of Economic Affairs and Employment of Finland 2022)	
**Interviewees**	
Official 7, Official 8	
**Engagement**	
*Activity*	*Related document(s) if available*
Survey in energy taxation working group	
Stakeholder hearing in energy taxation working group	Summary of the Hearings in the Working Group on Energy Taxation Reform (Ministry of Finance 2020)
Kick-off seminar for stakeholders and experts	Kick-off Seminar Presentation Materials
Stakeholder hearings in peat working group	Summary of the Hearings in the Working Group on Peat (Korhonen et al., n.d.)
Stakeholder hearings in sectoral integration working group	Summary of the Hearings in the Working Group on Sectoral Integration (Sektori-Integraatiotyöryhmän Väliraportti 29.1.2021 2021)
Climate policy round table	
Stakeholder meeting	Summary of Stakeholder Meetings on WEM and WAM Scenarios (Koljonen et al. 2021)
Statement hearings	Summary of the Statement Hearings (Huttunen et al. 2022)

We combined the interviews with document analysis to strengthen and validate the findings through triangulation (Bowen 2009). Data (e.g. policy documents) showing interactions between policymakers and engaged groups are valuable for identifying organisations’ views and examining how they influence policy processes (Gronow and Ylä‐Anttila 2019). The policy documents included the three key policy outputs and other documents related to public participation, including minutes, presentation materials, reports, and summaries of the results of citizen surveys and hearings (Table 1). The documents provided not only a good understanding of how public participation was carried out, but also contained information about citizen and stakeholder opinions.

We used NVivo for content analysis of the transcribed interviews and the documents. The interviews were coded deductively to identify objects, subjects, models, and orchestration of participation. Documents were read to extract information complementing the interviews. Based on document and interview data, an understanding of the participatory collective was formed. This participatory collective was then examined through the lens of the 4Ds of participation framework, identifying aspects related to dialogue, diversity, deliberation, and decision-making power. Documents were also analysed through this lens to supplement the information provided by the officials.

## Results

### Public participation in three policy processes

**Fig. 2:**
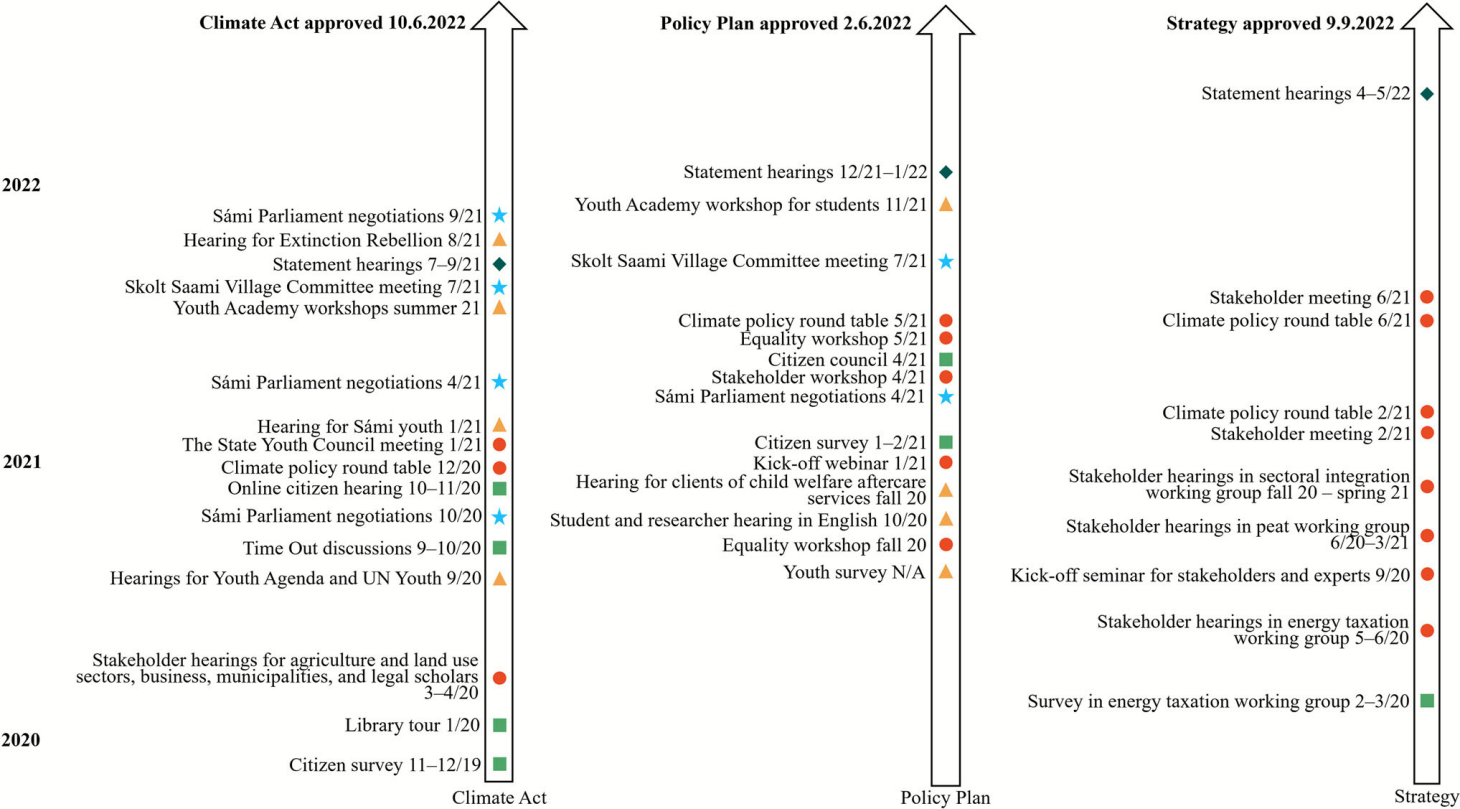
Timeline of participation in preparation of the Climate Act, Policy Plan, and Strategy. Shapes indicate the nature of participatory occasion. Diamond: statement hearings, circle: stakeholder or expert engagement, square: general public engagement, triangle: youth engagement, star: Sámi engagement

Finland’s climate policy is guided by the Climate Act. The new, second Climate Act came into effect in 2022. The Act establishes regulations for climate policy planning and monitoring, defines national climate goals (carbon neutral by 2035), and assigns responsibilities to authorities. Under the Climate Act, the climate policy planning system consists of several plans covering mitigation, adaptation, and land use (Climate Act 2022). One of these is the Policy Plan, prepared by the Ministry of the Environment. It outlines the measures for the non-emission trading sector to achieve the EU’s and Finland’s emission targets (Ministry of the Environment 2022). The planning system under the Climate Act runs parallel to the development of the Strategy, prepared once per electoral term by the Ministry of Economic Affairs and Employment. The Strategy includes the emissions trading, effort sharing, and land use sectors (Ministry of Economic Affairs and Employment of Finland 2022). All three policy processes complement one another and were prepared by government officials, with varying degrees of public and stakeholder involvement (Table 1, Fig. 2).

### Participatory collectives

#### The climate act

In the Climate Act, the core *issue* was to strengthen regulatory steering and ensure that Finland achieves carbon neutrality (Hallituksen Esitys HE 27/2022 vp Hallituksen Esitys Eduskunnalle Ilmastolaiksi 2022). Participation focused on discussing citizens’ wishes regarding the Act, its potential to achieve carbon neutrality, and gaining acceptability. In addition to climate neutrality, officials highlighted democracy-related objects, including the values of inclusivity, legitimacy, and transparency—suggesting that participation was also important in its own right, beyond contributing to the specific policy issue. They believed the Act “concerns everyone” (O2), and therefore citizens should be involved.

Regarding *the subjects of participation*, officials especially wanted to hear from those who are traditionally underrepresented in policy planning and vulnerable to climate change. Thus, the core target groups for public participation were young people and the Indigenous Sámi people.^1^ However, the preparation also aimed to involve ordinary citizens, and officials highlighted the importance of avoiding the impression that participants were handpicked. Officials believed this would make the preparation process more acceptable and prevent it from being labelled as "the Greens agenda" (O1) referring to the Green Party’s leadership in the Ministry.

*The models of participation* were top-down and invited. These included public surveys, hearings with stakeholders and youth, expert meetings, workshops and discussions with citizens, a statement hearing, and three negotiations with the Sámi Parliament. Participation events were held in various municipalities across Finland (Ministry of the Environment 2021d). Officials recognised their own influence on how public participation shaped up. As Official 3 put it, “*It's like with many things within administration, you know, it depends a lot on the individual official, whether something moves forward or how it is prepared*”. According to the officials, participation was intentionally more inclusive and deliberative compared to the preparation of the first Climate Act, due to increased public discussion on climate issues. Although public participation was invited, officials recalled that the idea to hear from UN Youth, Young Agenda, and Extinction Rebellion came from the young people themselves.

Regarding *the orchestration of participation*, enrolment was administration-driven. Officials stated that they made considerable efforts to make surveys visible through various channels, utilising social and traditional media. Local events were advertised in libraries and local newspapers. While generally open, specific events invited participants to ensure diversity, and stakeholder discussions involved a narrower group of recognised stakeholders. Some participation was also bottom-up: the Climate Move movement launched a campaign encouraging people to comment on the Act during the hearing phase, and 50 youth organisations submitted a statement. The Covid-19 pandemic necessitated the use of digital solutions. Additionally, the goals set for the legislative process were supported by participation materials in six different languages, including all Sámi languages spoken in Finland (Ministry of the Environment, n.d.-a). This unprecedentedly diverse participation, and the fact that the experience was new for officials, demanded extensive resources, at the expense of the officials' own health, resulting even in burnout, as Official 3 explained: "*the other side of the coin is that it's important to be realistic and not… it's not always necessary to strive for a ten-plus performance if you know that, for example, the project itself is already demanding. So, that's when officials become exhausted*”.

Despite the extensive public participation, officials reported a shared concern within the administration about the limited public knowledge of climate change. Furthermore, officials claimed that “the deep layers of society” (O4) were ultimately not reached through the engagement efforts, and that the aim of inclusivity encountered internal resistance. The preparing officials had hoped to produce Russian and Arabic versions of the materials, but their superiors considered it “out of question” (O2). Official 2 criticised the effectiveness of participation: “*I really tried to get those messages across to our team. I sent them to countless people within our own organisation, but no one seemed interested. It didn't seem to sink in.*” They also stated, they realised: "*oh damn, this Act doesn't match at all what the citizens have said*." (O2), as citizens had hoped for stronger enforceability of emission reduction targets (Ministry of the Environment 2021f), as well as better information and improved opportunities for participation in the preparation process (Ministry of the Environment, n.d.-a).

#### The policy plan

The Policy Plan investigates the actions needed to reduce emissions in the effort sharing sector to meet Finland’s climate targets (Ministry of the Environment 2022). Hence, *the issue of participation* was to discuss climate measures. According to officials, the *object of participation* was to strengthen the acceptability and justice of the planned measures. The officials “*wanted to test how citizens and stakeholders would respond to different measures*” (O6). Similarly to the Climate Act, public participation aimed for diversity in practices, consideration of sensitivities, and inclusion of the groups otherwise not engaged. The goal of acceptability was evident in the collection of information from citizens about potential negative impacts of policy measures. Officials highlighted the aim to increase transparency in the preparation and to give citizens more opportunities to influence.

*The main subjects of the participation* were citizens. Officials engaged elderly and disability councils from across Finland in equality workshops, along with Sámi and youth. Additionally, stakeholders and experts were heard, for example, in relation to agriculture and buildings. Officials felt they had achieved inclusive public engagement, although they acknowledged that those who did not feel a “great threat” or “great interest” (O5) in climate policy were unlikely to have participated. Furthermore, Official 6 noted: “*now perhaps these traditional stakeholder groups might feel they are not as strong anymore in this preparatory process as before, when we have expanded this scope to cover the whole society*” (O6).

*The models of participation* were initiated and organised by officials. As with the Climate Act, various channels were used. Already at the beginning, it was decided that ongoing relevant research and planning work would be linked to the preparation of the Policy Plan (The 1st Meeting of the Working Group Supporting the Preparation of the Medium-Term Climate Policy Plan (KAISU) 2020). The models of participation were chosen to “*ensure we hear all groups of people, even if they may not necessarily actively understand this opportunity or that this affects us*” (O1). The models included surveys, workshops, youth hearings, stakeholder meetings, a statement hearing, and a citizens’ jury organised in collaboration with a research project (Fig. 2).

Concerning *orchestration*, enrolment in participation took place through the ministry’s own communication channels: social media, web pages, and established meetings with stakeholders. Participants in the citizens’ jury were selected through random sampling (Kulha et al. 2021). The citizen survey received 18,000 responses, as information about it spread through the media. Officials considered it important to offer lightweight participation options: “*Especially these special groups—they don’t have time or the conditions to read our 200-page [policy plan] and comment on it. So, we have tried to make public participation in a way that there are opportunities for them to participate at their own level.*" (O5). In addition to researchers organising the citizens’ jury, young people were enlisted to help collect citizen views by high school students designing a survey (Ministry of the Environment 2022). An interpreter was present at meetings with the Sámi. According to officials, public participation was carried out as part of their everyday work and, “*partly it was a bit of an ex-tempore type, that we came up with along the way how things were handled*” (O6). Official 1 stated: “*it is important to keep public participation such it can be run as part of the everyday work*”, and “*we didn't make Mercedes or luxury-models like how we do things*”. Despite these efforts, some officials found the process too extensive and burdensome.

#### The strategy

The Strategy includes policy measures through which Finland is expected to meet the EU’s 2030 obligations and achieve the national 2035 climate target (Ministry of Economic Affairs and Employment of Finland 2022). Engagement in the Strategy was centred around three thematic working groups, where stakeholders and experts were consulted. The *object of participation* was to discuss the proposed measures and fulfil the formal requirement for public participation in the Strategy’s preparation. One of the officials admitted: “*primarily, engagement is done for the sake of engagement itself. It’s about demonstrating we have indeed engaged*” (O8). Officials acknowledged a general “trend” to involve more than just traditional groups and to assess the effects of policy actions on “*all possible and impossible parties*” (O8). Officials also recognised the goal of acceptability, viewing public participation as a means to foster more positive attitudes towards climate measures and facilitate their implementation, as participation increases participants' understanding of the policies and the processes behind them. Furthermore, they believed that participation could help expand and disseminate information to both policymakers and participants.

According to the interviewees, the key *subjects of participation* were various professional organisations directly affected by policy actions. Energy-related organisations were particularly prominent. Official 7 stated that in Finland, interest groups are not “crafty” but rather try to bring practical solutions to the table. Simultaneously, officials acknowledged lobbyists having better chances of achieving their goals through participation compared to ordinary citizens. Although stakeholder involvement remained central, it was broader than in the past: “*we really try to include everyone now*” (O7). For instance, environmental organisations and groups representing children submitted statements. The preparation process utilised expert knowledge and involved inter-ministerial collaboration, for example with the simultaneously prepared Policy Plan.

Regarding *the models of participation*, the process was administration-lead and invitation-based. These included expert-oriented seminars, webinars, working group sessions, public hearings, and open survey on energy taxation. Specifically, experts and lobbyists were invited to share their opinions and knowledge. Official 8 acknowledged that strong lobbying influenced the outcome regarding wind power and hydrogen. However, they also stated that the Strategy is fundamentally a “completed package” (O8), based on the previous versions. They believed further participation was unlikely to change anything, as it probably would not yield new insights.

Concerning *orchestration*, officials used email lists and press releases to disseminate information about participation opportunities. Officials noted that communication usually targets interested parties but could also reach new audiences with the same effort. Participation was mediated especially through various online participation and opinion platforms. Officials viewed information technology as enabling broader participation. Furthermore, they mentioned that participation often occurred as part of general meetings and other ongoing work. Media discourse on the topic was also used in the preparation process, and officials did not always consider separate participation arrangements necessary. Except for the digital statement platforms, officials acknowledged that citizens were not involved in the preparation process. They believed the Strategy was too broad and its topics too distant from everyday concerns to interest ordinary citizens. Official 8 stated: "*It's difficult to ask things from those who don't know them*". Furthermore, participation was limited in its ability to influence the outcome: “*Some aspects or a third of it or something are already decided in the previous year or in budget, and so on. They are just assembled here, so there is no real way to influence them anymore, other than saying it wasn’t good*” (O7).

### Outcomes of the participatory collectives

Evaluating the three participatory collectives using the 4D approach reveals both similarities and differences (see Table 2).

**Table 2:** Summary of the 4Ds by policy

	Climate act	Policy plan	Strategy
Description of the participatory collective	Invited youth and Sámi dialogue aimed at climate neutrality and acceptability	Invited citizen participation and new methods to identify acceptable climate actions	Official-led stakeholder discussions to verify details and information for policy preparation and fulfil participation requirements
Dialogue	Utilisation of two-way communication methods, such as library tour to gather diverse perspectives and inform citizens about the process	Multiple methods to inform citizens about policy planning process and involve everyone to gain understanding of diverse target groups and to make policy more transparent	Limited, one-sided citizen communication via online platforms and stakeholder discussions to ensure the technical details
Diversity	Efforts to recognise citizen perspectives (especially youth and the Sámi) through diverse methods, different language versions, possibility to express views beyond written statements, and regional events. Challenges included comprehensibility, low participation rates and resistance to multilingual options	Broad citizen involvement via various platforms and direct outreach aimed to include those typically excluded from traditional participation processes. Concerns remained about avoiding perceptions of bias or selective participation	While officials felt pressure to broaden participation to include non-traditional groups like young people and involvement of new groups was welcomed, citizens were seen to lack knowledge, and process was not considered from citizen perspective
Deliberation	Because emotionally charged and polarising nature of climate issues, officials recognised the need for diverse perspectives in the preparation process, and a careful focus on specific groups, rather than mixing totally different viewpoints	Climate policy was recognised to evoke strong opinions, prompting to provide information and expert access while navigating the risk of steering discourse in citizen jury and acknowledging survey respondents were often deeply invested in the topic	Participation was primarily seen as a tool for information dissemination and justifying pre-determined decisions, rather than a mechanism for deliberation
Decision-making power	Despite including Sámi-related provisions into the Act, citizen input was largely disregarded due to political manoeuvring and a reluctance to adopt legally binding measures	Economic considerations had to be prioritised alongside public input, emphasising non-punitive climate measures. Officials acknowledged the challenge of citizens grasping complex policy details, emphasising the need to clearly define engagement boundaries and manage expectations	Decisions remained heavily influenced by pre-existing frameworks, political agendas, budgetary constraints, and the influence of established lobbying groups, relegating citizen input to an obligatory formality

#### Dialogue

In the Climate Act and the Policy Plan preparations, officials invested in having a dialogue with citizens. Some long-term dialogue methods were used, such as a citizen’s jury in the Policy Plan preparation. Despite these efforts, one-way communication via websites, surveys, and hearings remained prominent and was considered important. The Strategy preparation process hardly involved citizens. Dialogue was present solely in stakeholder engagement, primarily to reassure that all technical details had reached the officials.

#### Diversity

The Climate Act and Policy Plan invested in new methods to reach diverse groups. Representatives of the Sámi were more strongly involved than before, and overall accessibility was considered by organising participation regionally, digitally, and in multiple languages. New methods and diverse involvement faced resistance, especially from officials’ superiors. The aim for diversity often failed in Climate Act preparation, with limited inclusion of the deep ranks of the public, youth struggling to understand the survey, and low attendance at events in Northern Finland. The participatory collective of the Strategy was more unilateral, focusing on stakeholders and expert knowledge. Officials justified this by citing the Strategy’s technical nature and citizens’ perceived lack of interest and understanding. However, there was little effort to make the complex issue more accessible. Still, Strategy officials welcomed the idea of involving new groups in the process.

#### Deliberation

Climate Act and Policy Plan officials believed that the preparation process should be informed by a wide range of perspectives requiring deliberation. Deliberation was most evident in the Policy Plan’s citizens’ jury, as the method itself is grounded in deliberative ideals. Officials perceived that climate policy evokes strong emotions, which increased participation and laid the ground for deliberation. Although some of the proposed climate actions received criticism from participants, citizens generally supported the policy outlines presented in the Climate Act and Policy Plan preparations, according to officials and policy documents; and participation did not produce polarisation. In the Strategy preparation, participation was largely viewed as a vehicle for disseminating information and justifying difficult decisions, with less emphasis on deliberation.

#### Decision-making power

Both the documents and interviews related to the Climate Act and Policy Plan suggest that participating citizens would have supported even more ambitious climate policies if the fairness of policy actions were ensured. Officials in these processes had difficulties conveying the results and messages from the participation forward. Citizens desired greater involvement, particularly at the outset of the process and for youth, as noted in the policy documents of Climate Act and Policy Plan. While efforts were made to engage young people, for example, a proposed youth climate panel was not established. Despite citizen support for the outlines of the Climate Act and Policy Plan, the final output no longer truly reflected the views expressed during the participation process, an official in Climate Act felt, as various changes were made during the political process. Still, the policies did incorporate elements that emerged during participation, especially regarding the Policy Plan. These were pleasing to politicians too, such as emphasising acceptability and carrots instead of sticks. Officials preparing the Policy Plan emphasised the importance of clearly defining the boundaries and realism of engagement to citizens. In the Strategy process, participation did not significantly influence the outcome. However, professional lobbying had a greater impact.

## Discussion

The main result of the analysis regarding the public participation is twofold. First, the public was involved in Finland’s climate policy participatory collectives to varying degrees, through diverse models and purposeful inclusion—particularly in the Climate Act and Policy Plan, but to a lesser extent in the Strategy. Second, although the participatory collectives were structured differently across policies and the public involvement varied within them, no participatory collective significantly granted decision-making power to citizens, and the engagement largely lacked influence. This meant that while citizens had opportunities to participate (and reason to believe that, by participating, they might have influence), the decisions were made elsewhere, and the outcomes of participation did not affect the decisions.

### Participatory collectives

Participatory collectives of the Climate Act and Policy Plan emphasised citizen participation, employing various models of participation and pursuing inclusiveness. These features were not evident in the participatory collective of the Strategy. We recognise two key reasons shaping climate policy collectives of participation, leading in involvement of the public and related differences.

Our research provides a snapshot of a situation in which not only the participatory collectives, but also the national climate policy were ‘in the making’ (Chilvers and Longhurst 2016, p. 586). This is evident, for example, in the fact that while previous studies emphasise a lack of public participation in Finnish climate and energy policies (Gronow and Ylä‐Anttila 2019; Kinnunen 2021), we identified greater public involvement, particularly in the preparations for the Climate Act and Policy Plan. Growing public interest in climate change, especially youth activism, has prompted political and ministerial levels to seek public opinion during preparation processes.

Although the government recognised the need for public involvement, it did not materialise evenly due to differences in how citizens were perceived. In the Climate Act and Policy Plan, officials recognised that citizens have the potential to influence the future, as Wamsler et al. (2020) express. In the Strategy, however, citizens were not considered to have the capabilities the participatory collective would require. The level and focus of each policy shaped participation. For example, when the Strategy addressed energy markets, actors from that sector were involved. It can be problematic when officials pre-determine who is considered to have something to contribute to the topic. All three policies contain themes that could resonate with citizens’ everyday lives, and despite recognising the topic’s complexity, the Climate Act and Policy Plan aimed to highlight the views of often excluded groups through various participation methods and orchestration. In the preparation of the Strategy, however, no effort was made to make the topic more approachable; instead, the relevant participants in participatory collective were considered to be experts and stakeholders. The differences in the responsible ministries for preparation are likely to have influenced this. Although society evolves, changing the culture of policy preparation can be challenging.

Officials played a significant role in participation by shaping how the participatory collective was formed, which models were chosen for participation, and who was included. Their role encompassed the power to facilitate participation by fostering dialogue, deliberation, and diversity. However, their authority generally did not extend to granting decision-making power to citizens.

### Positive and negative effects

The Climate Act and Policy Plan engaged diverse groups into dialogue, and even into deliberation, while the Strategy was less dialogical, deliberative, and diverse. However, all processes partially resulted in fake participation, understood in terms of decision-making power. This relates to two explanations.

First, experts and stakeholders had a strong role in participatory collectives, justified by the need for technical knowledge required to mitigate climate change (van Beek et al. 2024). Simultaneously, their opinions may have carried more weight than those of citizens. The influential inner circle can exclude alternative views and this way determine the outcomes of the policies (Gronow and Ylä‐Anttila 2019). Second, the objectives of participation were varied. They aimed to make the preparation more transparent, inform citizens, and increase the acceptability of climate policy. Thus, citizen impact on the outcome was not necessarily the main goal. Participation may have tacit agendas, such as testing new engagement methods or simply fulfilling the obligation of participation (Braun and Könninger 2018), as seen in the Strategy’s preparation. Thus, participation prioritised procedural acceptability through diverse models and target groups, rather than implementing the outcomes.

Overall, democracy can be undermined if the importance of participation is emphasised in a way that gives citizens the impression that their involvement automatically enhances their influence, regardless of how that influence is reflected in the outcomes. Emphasising procedural aspects can divert attention from effectiveness, aligning with Stirling’s (2008) observation that participation is often prioritised without ensuring its results are implemented. However, individual instances of public participation do not replace representative democracy, and it is important to avoid the opposite extreme, where too much power is given to a small group of people through participatory initiatives. The influence of public participation can also mean that it is duly considered in parliamentary processes, even if not implemented as such.

Another drawback of citizens' views not being visible in the outcome, despite their participation, relates to the inputs of participation. We found citizens would have preferred a more ambitious Climate Act than the one ultimately enacted. Although those who participated most actively were likely to hold strong opinions either for or against climate policies, other findings suggest that the public aims for slightly greater mitigation ambition than those in decision-making positions (Cattino and Reckien 2021). People in positions of power within government, business, and the economy tend to favour less ambitious proposals and may dismiss citizens’ transformative ideas. Thus, citizen involvement, and even deliberative processes, can ultimately fail to translate into sustained influence within a formal, top-down policymaking environment (Galván Labrador and Zografos 2024). However, participation does not always lead to stronger climate policies, even when citizens' messages get through to decision-making. This is because citizens do not always support strong climate policies, and officials and politicians seek outcome-based acceptability in addition to procedural acceptability. For example, the Policy Plan preparation addressed citizens’ concerns related to fairness, ending up with widely accepted but less effective policies. Overemphasising acceptability may lead politicians to avoid effective but controversial tools, thereby hindering climate action.

The impact of participation is not solely about influencing decision-making, even if the 4D approach emphasises it as one of the Ds. The effects of participation also emerge through the other Ds and occur at different levels, such as personal benefits or consultation (e.g. Fung 2006). However, when aiming for acceptability, it is crucial that—if it is implied that participation offers an opportunity to influence policies—then this should genuinely be the case. Otherwise, also the acceptability is at risk. The promises, expectations, and outcomes of participation must be aligned.

### Limitations and implications

We have examined public participation from the perspective of officials. More research is needed on citizens’ experiences to understand how they perceive their role in participatory collectives and the impact of their participation. It may be that citizens do not even prefer decision-making power (Perlaviciute 2022). Furthermore, the documents we focused on only enabled us to observe the uptake of the citizens’ recommendations and perspectives in the final outcomes. It would also be useful to look more closely into parliamentary processes related to the policies, to see how citizen’s input and roles were considered and discussed prior to the finalisation of the policies.

However, our approach has enabled us to understand how citizens can participate in and influence national climate policies from the perspective of officials and as visible in the documents. Our results are useful for further developing participation both in Finland and abroad. Democracies internationally are grappling with how to foster democracy through public participation in an impactful manner. This question is crucial for the credibility and resilience of democratic institutions at a time when they are being questioned. While our results concern climate policy, a similar analysis—combining systematic and normative perspective—could also be applied to other policy areas.

Our key contribution to public participation research is the demonstration that it is possible and beneficial to examine participation both systematically and normatively. A systematic approach reveals the multiplicity of participation, which can then be evaluated. Without a normative approach, the examination may remain incomplete and superficial, especially when the context is a vital policy process like national climate policy, where governance should be accountable for public input, as emphasised by the OECD (2024). The utilisation of our joint framework combining these approaches should be tested in the contexts of other countries and across different levels of governance.

The goal of Perlaviciute’s (2022) 4Ds is to increase the acceptability of climate policy. The approach does not prioritise any of the Ds over the others. Our article shows that the 4Ds are embedded in participatory collectives to varying degrees. The process may foster dialogue, diversity, and deliberation, but completely fail to redistribute decision-making power and only produce fake participation. Does this kind of participation matter? It meets a definition of participation as taking part and interaction, but not yet the political science perspective linked to democratic theory, which views participation as power-sharing (Carpentier et al. 2019). Our results highlight the importance of the impact of public participation for officials, as they regarded it as a failure when citizens’ perspectives were not reflected in the final policies. Furthermore, knowing that participation would not influence the decisions being made led some officials to question the value of public participation activities in the first place. Thus, decision-making power is important, and it can also facilitate the other benefits that public participation offers. These extend somewhat beyond the 4D framework and include aspects such as increasing awareness of climate change (Cattino and Reckien 2021) or raising public debate (Smith 2023). For example, in the case of the Climate Act and Strategy, participation attracted public attention, which in turn sparked people’s interest in getting involved. The impact can also extend beyond the specific policies in question: by elevating the climate crisis in political discourse and normalising participatory methods that can be scaled, for instance, to municipalities, as Smith (2023) demonstrates.

It should also be considered whether the emphasis on acceptability in both research literature and politics has become an overly dominant criterion for public participation in climate policy. While acceptability is important for democracy and for achieving climate goals, it cannot substitute for those goals themselves, as our article demonstrates. A strong commitment to democracy and to solving the climate crisis is needed.

## Conclusions

We examined public participation in the preparation of Finnish climate policy from an administrative perspective. Our findings reveal that the way participatory collectives were formed—through their objects, subjects, and models—and how they were orchestrated influenced how successfully they promoted dialogue, diversity, and deliberation.

While some preparation processes succeeded in generating positive effects that supported the acceptability of climate policies, none managed to empower citizens with decision-making power. We argue that participation cannot be deemed successful if it fails to redistribute decision-making power to citizens, despite fostering dialogue, diversity, and deliberation. The ability to grant decision-making power to citizens was limited due to perceptions of citizens’ capabilities; structural power imbalances, including stakeholder dominance; diverse objectives beyond citizen influence; and a focus on acceptability and process rather than impact and outcomes. Some of these obstacles are fundamentally beyond the control of officials, as policies are made under political direction. However, it would be important to reflect realistically on participation and to be open about it with citizens.

Our results suggest that, from a democratic perspective, instead of merely stating that participation exists and describing its form (the participatory collective), people doing public engagement should simultaneously consider its outcomes (the 4Ds) to assess the effects of participation. Further, it is necessary to analyse what qualifies as participation and what kind of participation is being aimed for: Participation for its own sake, for other impacts (e.g. raising public debate on climate issues), or for genuine citizen influence. For example, Dean’s (2017) typology, which highlights the diversity of public participation, could be helpful in this context. Especially within government, there should be transparency towards citizens so that they can assess the value of investing their time and energy in participation against their own goals and values—and, if necessary, choose other forms of civic activity that may have a more direct impact. Based on our findings, it is important for officials that participation is proportionate to the resources available, that public opinions collected through participatory processes are genuinely utilised, and that participation supports and benefits policy preparation. This highlights the importance of developing participation in a way that realistically meets its goals and fulfils its promises to citizens.

## Data Availability

The policy document data that support the findings of this study are publicly available and listed in the references. The interview data are available from the authors upon request.

## Footnotes

1 Sámi are the only officially recognised Indigenous people in Finland and EU. There are approximately 10,000 Sámi living in Finland and their traditional livelihoods are at risk due to climate change (The Sámi Parliament 2025).
